# Improving the Wear Resistance of Steel-Cutting Tools for Nuclear Power Facilities by Electrospark Alloying with Hard Transition Metal Borides

**DOI:** 10.3390/ma18215005

**Published:** 2025-11-01

**Authors:** Oksana Haponova, Viacheslav Tarelnyk, Tomasz Mościcki, Katarzyna Zielińska, Oleksandr Myslyvchenko, Kamil Bochenek, Dariusz Garbiec, Gennadii Laponog, Jaroslaw Jan Jasinski

**Affiliations:** 1 Department of Experimental Mechanics, Institute of Fundamental Technological Research Polish Academy of Sciences, Pawińskiego 5B, 02-106 Warsaw, Poland; tmosc@ippt.pan.pl (T.M.); kzielin@ippt.pan.pl (K.Z.); 2 Applied Material Science and Technology of Constructional Materials Department, Sumy State University, Kharkivska 116, 40007 Sumy, Ukraine; glaponog@ukr.net; 3 Technical Service and Industrial Engineering Department, Sumy National Agrarian University, H. Kondratiieva 160, 40021 Sumy, Ukraine; tarelnyk@ukr.net; 4 Physical Chemistry of Inorganic Materials Department, I. M. Frantsevich Institute for Problems in Materials Science, Omeliana Pritsaka 3, 04060 Kyiv, Ukraine; zvyagina47@gmail.com; 5 Department of Mechanics of Materials, Institute of Fundamental Technological Research Polish Academy of Sciences, Pawińskiego 5B, 02-106 Warsaw, Poland; kboch@ippt.pan.pl; 6 Łukasiewicz Research Network–Poznań Institute of Technology, Ewarysta Estkowskiego 6, 61-755 Poznań, Poland; dariusz.garbiec@pit.lukasiewicz.gov.pl; 7 National Centre for Nuclear Research, Centre of Excellence in Multifunctional Materials NOMATEN, A. Soltana 7, Swierk, 05-400 Otwock, Poland; jaroslaw.jasinski@ncbj.gov.pl; 8 National Centre for Nuclear Research, Materials Physics Department, A. Soltana 7, Swierk, 05-400 Otwock, Poland

**Keywords:** electrospark alloying, W–Zr–B electrodes, SPS, coatings, phase composition, microstructure, hardness, steel

## Abstract

This study focuses on improving the wear resistance of cutting tools and extending their service life under intense mechanical, thermal, and radiation loads in nuclear power plant environments. This research investigates the potential of electrospark alloying (ESA) using W–Zr–B system electrodes obtained from disks synthesised by spark plasma sintering (SPS). The novelty of this work lies in the use of SPS-synthesised W–Zr–B ceramics, which are promising for nuclear applications due to their high thermal stability, radiation resistance and neutron absorption, as ESA electrodes. This work also establishes the relationship between discharge energy, coating microstructure and performance. The alloying electrode material exhibited a heterogeneous microstructure containing WB_2_, ZrB_2_, and minor zirconium oxides, with high hardness (26.6 ± 1.8 GPa) and density (8.88 g/cm^3^, porosity < 10%). ESA coatings formed on HS6-5-2 steel showed a hardened layer up to 30 µm thick and microhardness up to 1492 HV, nearly twice that of the substrate (~850 HV). Elemental analysis revealed enrichment of the surface with W, Zr, and B, which gradually decreased toward the substrate, confirming diffusion bonding. XRD analysis revealed a multiphase structure comprising WB_2_, ZrB_2_, WB_4_, and BCC/FCC solid solutions, indicating the formation of complex boride phases during the ESA process. Tribological tests demonstrated significantly enhanced wear resistance of ESA coatings. The results confirm the efficiency of ESA as a simple, low-cost, and energy-efficient method for local strengthening and restoration of cutting tools.

## 1. Introduction

Metal-cutting tools (MCTs) are essential for the effective mechanical processing of materials in a variety of industries. The high performance, precision and reliability of these tools directly impact the quality of equipment manufacturing and repair, thereby influencing the economic efficiency of enterprises. MCTs are particularly important in the production and maintenance of high-tech systems, which require greater precision and stability in processing.

Mechanical processing is widely used in the automotive, aerospace, defence, medical, electronics and other industries. The use of MCTs in nuclear energy is of particular interest, especially during the repair and dismantling of nuclear power plants (NPPs) [1,2,3].

The use of tools in elevated radiation environments presents significant challenges. Materials and processing technologies must be adapted. During repair work in radiation zones, tools are subjected to mechanical, thermal, and ionising radiation loads, which can alter their properties over time. This requires enhanced radiation resistance, thermal stability, and reliability. Tools must meet stringent demands, including high wear resistance, heat resistance, resistance to radiation-induced degradation, and thermal stability to prevent deformation. They must also be adaptable to remote control or automated systems, which is critical in areas with elevated radiation backgrounds.

In such conditions, tools must be made to last. This is achieved by using materials with high hardness and heat resistance. Some of the materials used include high-alloy tool steels, hard alloys, ceramics and superhard composite materials [4,5]. Functional coatings are widely used to reduce the coefficient of friction and prevent thermal wear [6,7,8]. Thus, improving the MCTs used to repair parts that operate in conditions of radiation exposure is a technical and strategic task. It enhances nuclear safety and equipment reliability. Developing tools that can withstand extreme conditions improves the effectiveness of repair work and extends the service life of critical NPP equipment.

During the dismantling and repair of equipment used in nuclear power facilities, particular attention is given to selecting MCTs that can operate effectively in conditions involving an increased radiation background, temperature loads, and contact with activated structural materials. The majority of research focuses on improving tool materials, processing methods, and creating multifunctional, wear-resistant coatings.

Traditionally, high-speed steels (HSSs) containing tungsten, molybdenum, vanadium, chromium have been used as the main material for manufacturing MCTs. These steels offer a balance of hardness and toughness, maintaining mechanical strength at temperatures of up to 650 °C. This makes HSSs suitable for tools operating at moderate speeds, particularly in intermittent cutting conditions or when machining complex surfaces. HSSs with special coatings are now preferred due to limited wear resistance of cast steels. Nitrides, carbides, borides, oxides are among the most common coating materials used to improve the performance of cutting tools. The most widely researched and industrially implemented coatings include titanium nitride (TiN), titanium carbide (TiC), titanium carbonitride (TiCN), aluminium oxide (Al_2_O_3_), titanium boride (TiB_2_), diamond-like carbon (DLC), and boron nitride (BN), and composite multilayer structures such as CrAlSiN (CrAlBN). These coatings significantly improve wear resistance, microhardness, thermal stability and corrosion resistance [9,10,11,12,13].

Hard alloys with a cobalt matrix remain a widely used class of cutting material in industry. They are extremely hard, with a hardness of 1400–1600 HV, and highly heat-resistant, with a melting point of 900–1000 °C. Cobalt forms long-lived radioactive isotopes, creating a demand for alternative, cobalt-free tool materials that offer high mechanical properties while minimizing radioactive isotope formation [14].

In response, research into alternative coating materials has been actively pursued. Focus areas include tungsten-based compounds with ferromagnetic or nickel bonds (WC–Fe, WC–Ni, WC–FeNi) [15]; titanium and boronitride matrices (TiC–FeCr, Ti(C,N)–Fe, cBN–Al–TiN) [16,17]; ceramics (A_l2_O_3_, Si_3_N_4_, ZrO_2_); and super-hard materials (PCBN, PCD) [18,19,20,21].

Due to its high density, tungsten exhibits excellent gamma radiation shielding [22]. However, pure tungsten is less efficient as a neutron absorber. Adding boron enhances neutron protection, while transition metal borides, particularly tungsten borides, combine high hardness, thermal and chemical stability, and effective gamma/neutron shielding [23,24,25,26,27].

Alloying tungsten borides with tantalum, niobium, or zirconium increases hardness and improves plasticity, fracture toughness, and thermal stability above 650 °C, even under thermal shock [28,29,30]. Tungsten borides doped with zirconium are promising for protective coatings. They can be synthesized via spark plasma sintering [31,32], RF magnetron sputtering [33], RFMS–PLD [34], and HiPIMS [35]. Coatings of W_0.84_Zr_0.16_B_2.5_ on Inconel 617 showed high hardness, though helium ion implantation reduced hardness due to bubble formation and swelling.

Studies of W–Zr–B thin films revealed a nanostructured microstructure, high hardness, high elastic modulus (~270 GPa) and stable substrate adhesion [33], retaining mechanical properties at elevated temperatures and in aggressive environments. This makes them suitable for cutting tools in dismantling, decontamination, and nuclear reactor repair operations. However, these methods face practical limitations in production due to equipment requirements, deposition rates, and complex geometries [35].

The technology of electrospark alloying (ESA) becomes particularly significant. It is a pulsed surface modification method involving short-term, localised energy transfer between the electrode and the substrate via an electric spark. This pulsed effect results in local melting, ultra-fast cooling, and the formation of dense, nanostructured, and amorphous layers that adhere strongly to the substrate. ESA is used to successfully deposit solid phases, particularly TiB_2_, ZrO_2_, CrN, WC and FeNiCrB, which significantly improve the mechanical, tribological and corrosion properties of tool materials [36,37,38]. ESA also offers practical advantages in challenging production environments: it can strengthen areas with limited access, essential for repairs. Compared to other coating methods, ESA stands out for its cost-effectiveness, energy efficiency, environmental friendliness, mobile equipment and straightforward process control [39]. This makes it highly relevant for rapid restoration or strengthening of tool surfaces without dismantling equipment.

Despite research on boride- and tungsten-based materials and coatings, W–Zr–B prepared via ESA remains largely unexplored. ESA enables rapid, localized deposition of dense layers on complex geometries, which is difficult with conventional methods. This technology combines the high hardness, thermal stability, and corrosion resistance of the coatings with practical advantages such as cost-effectiveness, mobility, and suitability for in situ repair of nuclear equipment. Therefore, developing W–Zr–B coatings using ESA is crucial for improving tool performance in nuclear applications.

The aim of this paper is to develop a hard and therefore wear-resistant, functional coating based on the W–Zr–B system. This will be synthesised using the spark plasma sintering (SPS) method and deposited on a metal-cutting tool made of high-speed steel (HSS). The preparation of coatings will be carried out using electrospark alloying in ambient air. The resulting layers will then be researched in terms of their microstructure, phase composition and operational characteristics. This will take into account the requirements for tools used in repair and dismantling work at nuclear power facilities.

## 2. Materials and Methods

Using a Turbula^®^ T2F shaker-mixer (WAB, Muttenz, Switzerland) and the mixtures of 74.1 wt% tungsten (99.9% pure, APS: 25 μm), 11.6 wt% zirconium (99.8% pure, APS: 250–350 μm), and 14.3 wt% amorphous boron (95%) powders were mixed for 30 min. An HP D 25/3 (FCT Systeme, Frankenblick, Germany) furnace was used to sintering the resulting powder mixtures under vacuum. The parameters of the SPS process were earlier described in details in [40] and were: sintering temperature 1650 °C, heating rate 200 °C/min, holding time 25 min, compacting pressure 50 MPa. There were disks made that were two inch (~50 mm) in diameter and about 3.5 mm thick. The sintered targets were then cut by the electro-discharge machining (EDM) method to obtain the electrodes for ESA process.

The specimens obtained by SPS method was measured by Archimedes method to obtain the real density. Scanning electron microscope in back scattering electron mode (SEM-BSE) was used for microstructure/composition observation. The exact chemical composition was measured with Energy Dispersive Spectroscopy (EDS) with 10 kV accelerating voltage.

HS6-5-2 steel was used as the substrate, the chemical composition of which is presented in Table 1. The samples were heat-treated after being hardened at 1200–1240 °C with oil cooling, then tempered at 540–570 °C to achieve a hardness of 64–67 HRC. The samples had dimensions of 10 × 10 × 10 mm. The dimension of electrodes were: diameter 4 mm, length 20 mm.

Electrospark alloying was performed on the “Elitron 52-A” (Experimental Plant of the Institute of Applied Physics, Academy of Sciences of Moldova, Chisinau, Moldova) unit in manual mode at discharge energies of 0.36 and 0.90 J, ensuring surface alloying productivity at a level of 1.0–1.3 cm^2^/min (Table 2). Additional treatment was applied using the same electrode and reduced discharge energy (Wp = 0.05 J) to reduce surface roughness after ESA.

**Figure 1:**
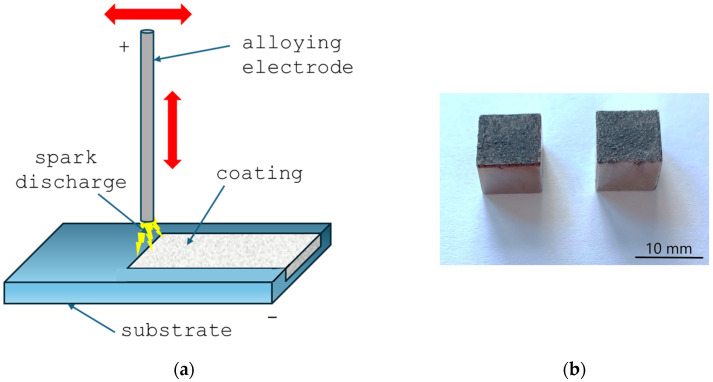
ESA process scheme (**a**) and samples after treatment (**b**). Red arrows indicate the movement of the electrode.

Figure 1 shows a scheme of the ESA process and photographs of the samples after treatment, illustrating their appearance after treatment. Three samples were fabricated and tested for each ESA mode.

The surface roughness was examined using a Talysurf stylus scanning profilometer (Taylor Hobsson, Leicester, UK), in accordance with the standard method. This enabled roughness profiles to be recorded, from which the arithmetic mean deviation from the mean line was calculated. To obtain adequate statistics, five measurements were performed on each sample. Definitions and parameters for the determination of surface texture by profile methods are compliant with the standard ISO 21920-2:2021. Surface roughness was determined as the mean of five measurements per sample.

The surface morphology and cross-sectional microstructure of the obtained samples were examined using a scanning electron microscope (SEM, JSM-6010PLUS/LV, JEOL, Akishima, Japan), and the elemental composition of the coatings was analyzed by energy-dispersive X-ray spectroscopy (EDS) to evaluate diffusion processes and elemental distribution within the modified layer. For microstructural analysis, the specimens were sectioned transversely and prepared using standard metallographic procedures, including sequential grinding, polishing and etching [41]. Metallographic tests were conducted using stereometric metallography methods.

The phase composition was examined using X-ray diffraction (XRD) with a AXRD Benchtop diffractometer (PROTO Manufacturing Ltd., Windsor, ON, Canada) using CoKα radiation lamp (λ = 0.17902 nm).

In order to examine changes in hardness under the influence of the processes carried out, a Wilson VH1102 microhardness tester (Buehler, Lake Bluff, IL, USA) was used. Measurements were performed on the cross-sections of the samples using the Vickers method. The maximum applied load was 20 g. Hardness was measured at five different points on each of three samples, and the average value and standard deviation were calculated.

Wear resistance was evaluated using a Micro Combi Tester MCT3 (Anton Paar, Corcelles-Cormondrèche, Switzerland) by performing a reciprocating wear test. An Al_2_O_3_ corundum ball with a diameter of 6 mm was used for the test, which moved cyclically across the surface of the sample. A constant normal load of 5 N was applied to all specimens. The ball traversed the surface at a linear speed of 600 mm/min, with a total sliding distance of 10 m.

## 3. Results and Discussions

### 3.1. The Structure and Properties of Electrode Material

**Figure 2:**
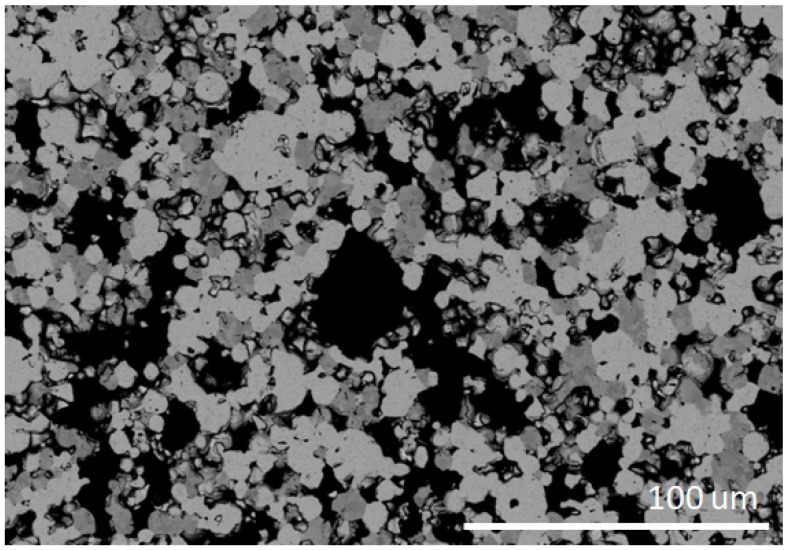
Scanning electron microscope in back scattering electron mode (SEM-BSE) observation of SPS-ed W-Zr-B targets microstructure.

**Figure 3:**
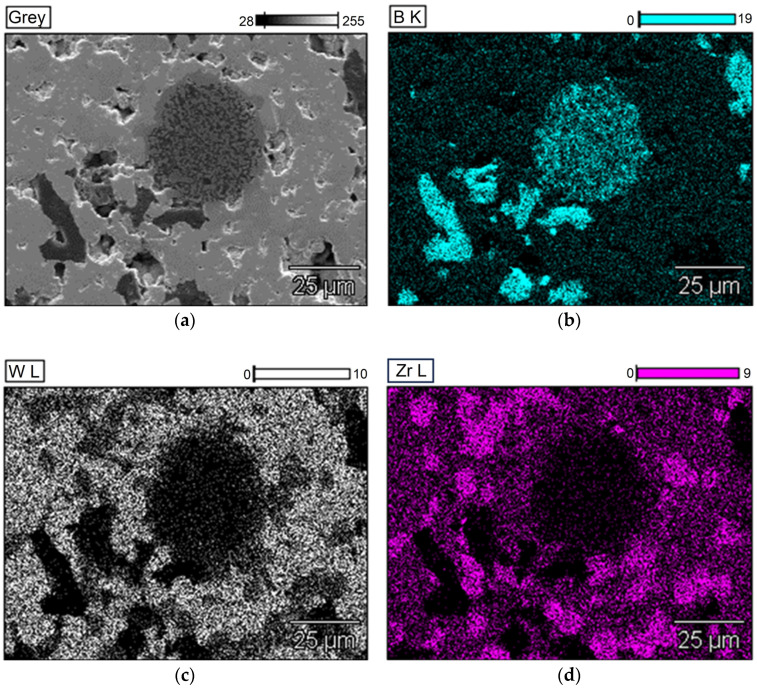
EDS maps of exemplary SPS-ed target sample (W_0.76_Zr_0.24_B_2.5_) (**a**) SEM micrographs, (**b**) boron, (**c**) tungsten, (**d**) zirconium distribution.

Figure 2 shows an SEM-BSE image of a fragment of a W-Zr-B disc sintered using SPS. Since the powders were not ground but only mixed using the Turbula^®^ T2F shaker-mixer that operates on Schatz’s geometric theory, employing a three-dimensional motion (rotation, translation, and inversion) to mix materials in a contained vessel, then the shape of the grains corresponds to the shape of the powders used. At the same time, a clear difference in the contrast between individual grains is visible. Because tungsten has the highest atomic mass, areas with a high content of this element appear the brightest in SEM-BSE images. In the case of boron, the grains are the largest and appear black. The third colour (dark gray) represents areas with a high zirconium content. This indicates that the discs contain areas where amorphous boron, which has not reacted with the transition metals (TM) tungsten and zirconium, can be observed. This may be due to the fact that a surplus of boron was used for sintering compared to the stoichiometric content, i.e., TMB_2.5_ instead of TMB_2_. The number of fields with the tungsten-rich phase is significantly greater than with the zirconium-rich phase. This is due to the number of individual elements, i.e., Zr/Zr + W = 0.24. It should be noted that using this composition and similar sintering conditions, Garbiec et al. obtained sinters with the highest hardness, i.e., 26.6 ± 1.8 GPa. In addition, this material showed electrical conductivity up to 3.961∙10^6^ S/m, which is similar to cemented carbides WC–Co [40]. The conclusions obtained from the analysis of SEM-BSE images are confirmed by the EDS analysis presented in Figure 3.

The density of the obtained discs determined by the Archimedes method is 8.88 g/cm^3^, which in comparison to the theoretical value of 10.43 g/cm^3^ determined for the W_0.75_Zr_0.25_B_2_ compound with the P6_3_/mmc (194) structure [33] gives a porosity of the order of 15%. Considering that the sinters were made with an excess of 2.5 of (TMB_2.5_) boron instead of 2 (TMB_2_), and the theoretical calculations used a stoichiometric value of 2, the theoretical density values are overestimated due to the atomic mass of boron (10.8) being significantly lower than the masses of the other components. Reducing the theoretical density also reduces the porosity below 10%.

**Figure 4:**
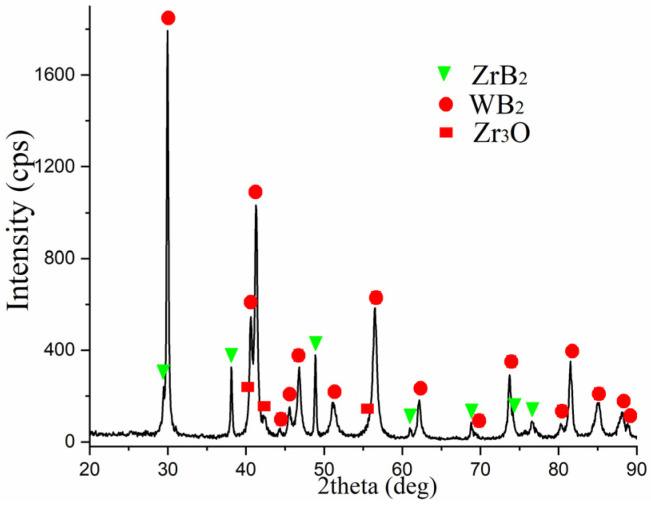
X-ray diffractograms of the alloying electrode.

The XRD analysis presented in Figure 4 shows that in addition to the WB_2_ phase (P6_3_/mmc), ZrB_2_ (P6/mmm) is present in the sinters. However, no peaks from crystalline boron were observed, confirming that the grains of this element observed in Figure 2 and Figure 3 did not crystallize during sintering. Theoretical calculations performed by Mazdziarz et al. show that a stable W_0.76_Zr_0.24_B_2_ phase may exist [33]. This is also confirmed by analysis of chemical composition maps, where zirconium is also present in places where tungsten occurs. However, since the solubility of zirconium in the WB_2_ crystal lattice is limited and the solubility limit as determined by PXRD is 10 at.% for Zr [29], the use of 24 at.% Zr relative to tungsten also allows for the synthesis of crystalline ZrB_2_. Despite their high hardness, the obtained discs are coherent and do not crumble, which allowed them to be used to obtain electrodes for ESA.

According to the obtained data, the main phase is tungsten diboride (WB_2_), with lattice parameters *a* = 2.983 Å and *c* = 13.879 Å, and the sample also contains a hexagonal phase of zirconium diboride (ZrB_2_) with lattice parameters *a* = 3.165 Å and *c* = 3.520 Å. Additionally, due to the high temperatures reached during sintering, a small amount of zirconium oxide (Zr_3_O) was formed, with lattice parameters *a* = 5.6295 Å and *c* = 15.5925 Å.

Quantitative phase analysis showed that the main phase, WB_2_, accounts for around 72 wt%. The ZrB_2_ phase accounts for around 23 wt%, while the zirconium oxide (Zr_3_O) content does not exceed 5% by wt. (Table 3).

### 3.2. Study of Surface Topography of Samples After ESA

**Figure 5:**
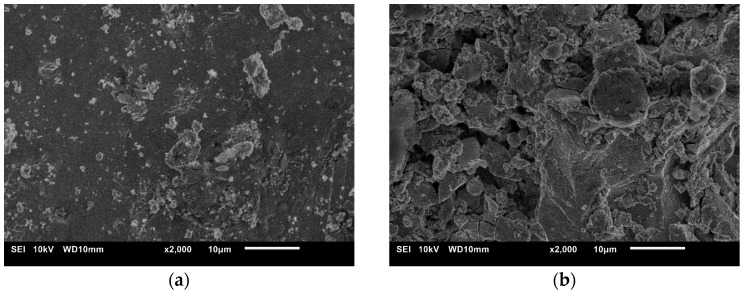
Coating morphology on HS6-5-2 steel after ESA at discharge energies: (**a**) 0.36 J, (**b**) 0.90 J.

**Figure 6:**
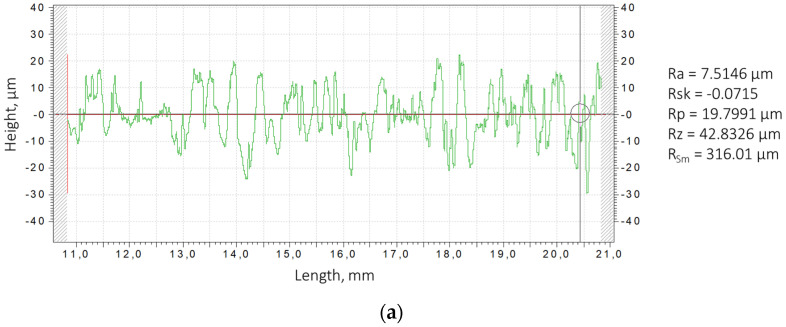

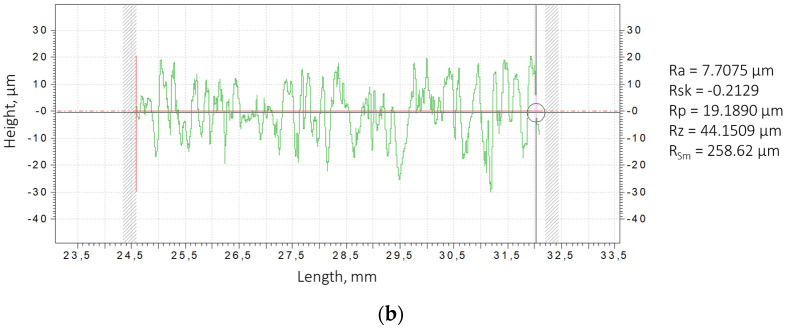
Surface roughness profilograms for steel HS6-5-2 samples after the ESA at discharge energies: (**a**) 0.36 J, (**b**) 0.90 J.

Analysis of the surface topography of samples after ESA showed that its characteristics depend significantly on the treatment mode, particularly the discharge energy. At low energies (0.36 J), a relatively uniform, thin layer with small microprotrusions forms (see Figure 5a). This surface has relatively low roughness, which is associated with the limited volume of molten metal involved in the transfer and rapid crystallisation process (see Figure 6a).

As the discharge energy increases, the thermal effect on the microvolume of the surface intensifies, resulting in melting and even local evaporation of the substrate and electrode material. This results in deeper craters and a developed micro-relief structure. During rapid cooling, some of the molten metal droplets are ejected beyond the discharge zone, while others solidify to form uneven layers. This leads to an increase in the height of micro-irregularities, and consequently an increase in roughness parameters (see Figure 5b).

Considering that after ESA samples at discharge energies of 0.36 J and 0.90 J possess high roughness, additional processing was performed at Wp = 0.05 J. The roughness indicators after these treatments for both modes remain similar: Ra = 7.5146 µm at Wp = 0.36 J and Ra = 7.7075 µm at Wp = 0.90 J. Despite the similar Ra values, noticeable changes in surface topography reflect the mechanism of coating formation by the ESA method, including differentiated material accumulation and formation of a special structure (Figure 5).

The effect of roughness on the performance of metal-cutting tools is ambiguous. Increased roughness can negatively affect geometric accuracy, wear resistance, cutting edge stability and contribute to stress concentration and the formation of microcracks. However, a more developed surface can provide an increased contact area, which contributes to the retention of lubricating and cooling media in the cutting zone. This has a positive effect on the thermal regime of cutting and reduces wear intensity.

In view of this, it is advisable to carry out additional processing operations after electrospark alloying, such as grinding, surface plastic deformation methods, laser processing, magnetron sputtering, etc. These operations allow roughness parameters to be optimised and a functionally effective surface layer to be formed.

As shown in papers [39,42,43], ESA-based hybrid technologies offer a synergistic effect by combining the high hardness and wear resistance of hardened layers with the controlled roughness and increased durability of parts.

### 3.3. Microstructural Analysis of ESA Coatings

**Figure 7:**
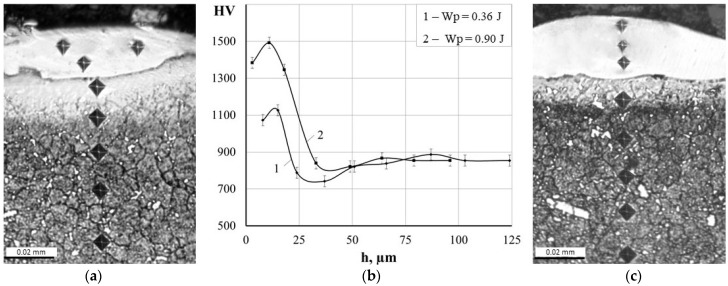
Microstructures (**a**,**c**) and microhardness distribution (**b**) of coatings after ESA at discharge energies: (**a**) 0.36 J, (**c**) 0.90 J.

Figure 7 shows the microstructure of the samples after ESA under different conditions, as well as the microhardness distribution in the obtained coatings.

Metallographic analysis revealed the presence of a so-called ‘white layer’ [43], which is characteristic of electrospark coatings and not susceptible to chemical etching. Depending on the treatment mode, its thickness ranges from 10 to 30 μm (see Table 4). The surface layer exhibits a maximum microhardness of 1125.9–1492.1 HV, which is similar to values characteristic of electrodes synthesised by the SPS method. The obtained hardness values are much higher compared to coatings in the form of Fe-TiB_2_ composites obtained by the TIG method (606 HV_0.1_) [44] or WC-Fe composite coatings fabricated by the laser wire cladding method (577.9 HV_0.2_) [45].

During ESA, in addition to the transfer and deposition of electrode material on the surface, complex physicochemical processes occur. The studies [46,47] shows that during electrospark treatment of iron-based alloys, there is a significant reduction in the size of substructure blocks, an increase in defect density, and an increase in micro-distortions in the heat-affected zone. Similar processes may occur with the materials under study, which could explain the formation of a ‘white layer’ with high hardness.

A diffusion zone is located beneath the hardened surface layer that forms during the ESA process. Due to localised heating within the intercritical temperature range (i.e., between Ac1 and Ac3 (Acm)), this zone experiences partial (incomplete) phase reformation and intensive diffusion of alloying elements. Consequently, a transition zone with a modified microstructure is formed, characterised by partial dissolution and redistribution of carbides, a small proportion of austenitised areas, and a relatively fine-grained structure following rapid cooling [48].

As can be seen in Figure 7b, the microhardness of the diffusion zone decreases gradually from the upper (‘white’) layer to the metal substrate. This microhardness gradient indicates a gradual change in properties and demonstrates the presence of a strong adhesive (metallurgical) bond between the coating and the substrate. This reduces the risk of delamination under load and promotes a more uniform distribution of stresses.

**Figure 8:**
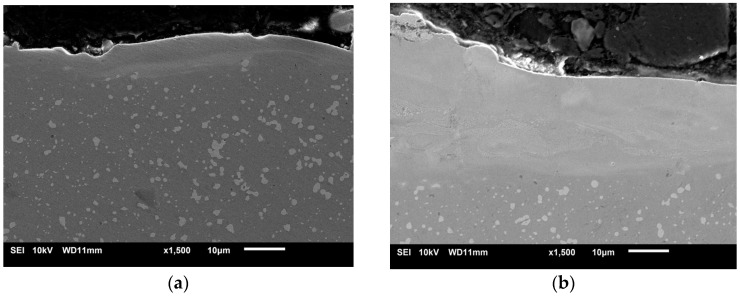
Structures of coatings after ESA at discharge energies: (**a**) 0.36 J, (**b**) 0.90 J.

SEM analysis enabled the surface layer structure to be visualised after ESA (see Figure 8). The images show a multilayer structure comprising an upper doped layer with pronounced uneven morphology, a transitional diffusion zone and the underlying metal substrate. The upper layer shows the hardened craters and irregularities that are characteristic of electrospark treatment. In contrast, the diffusion zone has a denser, more homogeneous structure, with signs of partial phase reformation. The boundary between the coating and the substrate is blurred and free from cracks or delamination, which confirms the formation of a strong adhesive bond. Additionally, microphotographs reveal fine-grained carbide inclusions distributed in the near-surface region, consistent with the results of optical microscopy and microhardness testing.

To analyse the diffusion processes that occur during ESA, the elemental composition of the coating obtained at a discharge energy of Wp = 0.90 J was tested at successive depths in 5 µm steps (see Table 5). The results demonstrate that the surface layer is enriched in the main components of the electrode material: tungsten (up to 57.46 wt%), zirconium (up to 11.69 wt%) and boron (up to 5.78 wt%). Additionally, an elevated carbon presence was identified near the surface, which is probably a consequence of carbon absorption from the atmosphere, given that the ESA process was conducted in air. With increasing depth, the concentration of these alloying elements gradually decreases. They approach the nominal composition of the HS6-5-2 steel substrate. The detection of substrate elements (Fe, Cr, Mo, V and Ni) in the coating demonstrates that partial melting and intermixing of the electrode materials occurred due to the high pulsed temperatures generated during ESA. This results in the formation of a diffusion-strengthened layer with a graded elemental distribution, enhancing the adhesion and overall performance of the coating.

### 3.4. X-Ray Analysis of ESA Coatings

**Figure 9:**
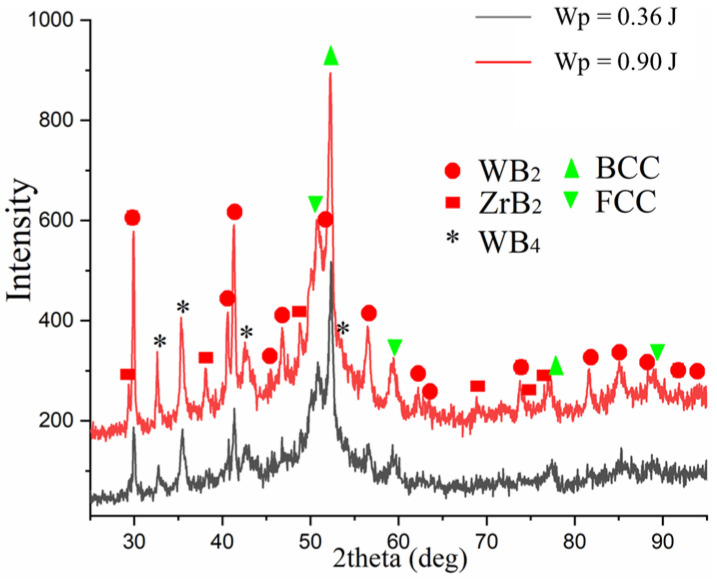
X-ray diffractograms of ESA coatings obtained at different discharge energies.

The phase composition of the obtained coatings is more complex than that of the electrode used for deposition (see Figure 9). In addition to the ZrB_2_ and WB_2_ binary borides characteristic of the electrode obtained at Wp = 0.36 J, high-boron WB_4_ phases appear in the surface layer. This indicates more intense mass transfer of the electrode material and diffusion of boron, which becomes saturated in the near-surface zone.

Additionally, phases with BCC and FCC lattices were detected, which may be the result of intensive mixing of the substrate material and the alloying electrode, the formation of iron-based solid solutions alloyed with tungsten and zirconium, as well as phase transformation products. These products are formed during rapid cooling of the molten layer in the cooling process after ESA, which is typical for highly concentrated pulsed energy sources.

Thus, an increase in discharge energy results in a transition from relatively simple boride and oxide phases to a more complex, multiphase system involving the formation of solid solutions and high-boron compounds. This indicates an intensification of diffusion processes and an increase in chemical activity in the discharge zone. This can positively affect the mechanical properties of coatings.

### 3.5. Wear Resistance of ESA Coatings

Reciprocating wear tests were performed to evaluate the wear resistance of the obtained coatings. To reduce surface roughness and stabilise the surface microrelief, the samples were subjected to grinding after ESA. The surface roughness values are presented in Table 6.

**Figure 10:**
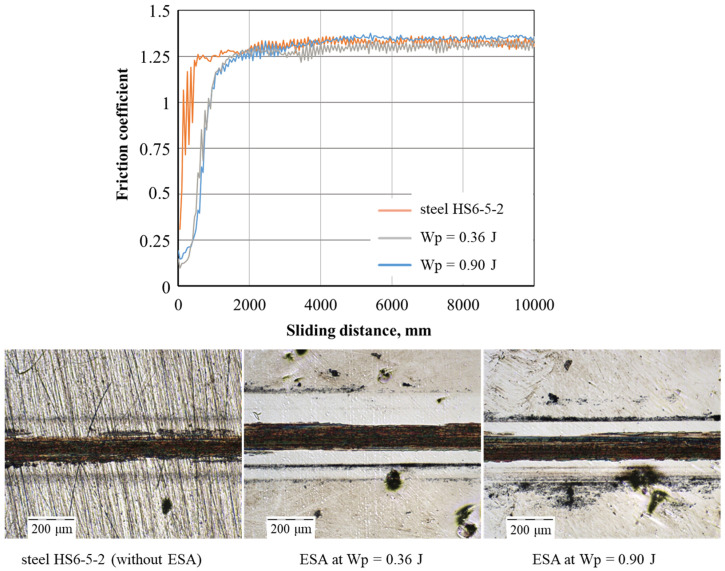
Tribological behaviour of ESA coatings.

Figure 10 shows the variation in friction coefficient with increasing sliding distance, as well as the wear traces after the reciprocating abrasion test. The friction coefficient for the substrate (HS6-5-2 steel) and the coated samples obtained using the ESA method is approximately 1.2. There is little difference in the friction coefficient between the steel and coatings. This similarity is due to the formation of a stable tribological layer (tribofilm) at the interface where the indenter and the sample come into contact during sliding. This layer equalises the surface energy of the contact area, providing similar dry friction conditions. Furthermore, coatings produced by the ESA method exhibit increased microhardness and a specific structure, which restricts plastic deformation during friction and minimises the risk of adhesive wear.

The results obtained correlate well with the observed morphology of the wear tracks (see Figure 10). Following the abrasion test, the wear traces on the treated samples were barely visible, with only the peaks of the surface asperities remaining. This demonstrates the high resistance of the coatings to abrasive wear and their consistent tribological performance under dry contact conditions.

The wear depth was calculated based on the volume of material removed by frictional forces per unit area. Table 7 shows the average values obtained, taking measurement uncertainty into account. For HS6-5-2 (HSS) steel, the average wear depth was approximately 1.5 μm. By comparison, a significant reduction in wear intensity was observed after grinding the surface formed by ESA: the average wear depths were 0.88 ± 0.33 μm and 0.69 ± 0.03 μm, respectively, at Wp = 0.36 J and Wp = 0.90 J.

These results suggest that ESA coatings not only exhibit increased microhardness, but also significantly higher wear resistance. This has important practical implications, as it extends the service life of tools, improves their operational reliability and reduces maintenance costs.

## 4. Conclusions

1. This research confirmed that W–Zr–B system materials are promising for use as coatings for cutting tools designed to operate under conditions of increased mechanical, thermal and radiation loads. These coatings hold promise for use in repair and installation operations at nuclear power facilities. Moreover, W-Zr-B ceramics are characterized by good electrical conductivity, which allows for the effective use of electrospark alloying (ESA) for the preparation of coatings.
2. The studied electrode materials of the W–Zr–B system, synthesised by the SPS method, are characterised by a heterogeneous microstructure with areas enriched with tungsten, zirconium and excess boron. X-ray analysis confirmed the presence of the main phases WB_2_ (~72% by mass) and ZrB_2_ (~23% by mass), as well as a small amount of zirconium oxide (~5% by mass) formed as a result of high-temperature sintering. The samples obtained are characterised by high hardness (26.6 ± 1.8 GPa), density (8.88 g/cm^3^) and rationally low porosity (<10%). The preservation of mechanical integrity and the absence of brittleness indicate the high structural stability of the material and confirm its practical application as electrodes for ESA.
3. Coatings on HS6-5-2 tool steel obtained by ESA under different processing conditions were studied. Surface topography analysis showed that increased discharge energy forms a more developed micro-relief with craters and micro-protrusions. Despite similar Ra values (~7.5–7.7 μm), the nature of irregularities varies, reflecting the ESA coating formation mechanism and indicating opportunities for further optimization using hybrid techniques.
4. Metallographic analysis revealed the formation of a hardened surface layer (“white layer”) with a thickness of 10–30 μm and microhardness of 1125.9–1492.1 HV. A diffusion zone beneath the layer shows a gradient decrease in microhardness to the substrate, indicating strong metallurgical bonding without cracks or delamination. Elemental analysis confirmed that the coating is enriched with the main components of the alloying electrode material: tungsten, zirconium and boron. These elements decrease in concentration with depth, forming a diffusion-strengthened transition layer.
5. Coatings after ESA have a more complex phase composition compared to the electrode. In addition to ZrB_2_ and WB_2_, WB_4_ phases and BCC/FCC solid solutions form due to diffusion and intensive mixing during ESA. Higher discharge energy leads to multiphase layers with high-boron compounds, reflecting intensified diffusion and chemical activity.
6. ESA coatings demonstrated superior wear resistance compared to the uncoated HS6-5-2 steel substrate. The test results showed a significant reduction in wear depth for the ESA-treated samples, with average values of 0.88 ± 0.33 μm (Wp = 0.36 J) and 0.69 ± 0.03 μm (Wp = 0.90 J), compared to 1.52 ± 0.12 μm for the uncoated steel, while the friction coefficient remained approximately 1.2.
7. The results demonstrate ESA is a simple, portable, and energy-efficient method for local strengthening of cutting tools, producing coatings with high adhesion, increased hardness, and overall high quality.
8. Further development of this work should be directed towards optimising hybrid technologies based on ESA (surface plastic deformation, laser treatment, magnetron sputtering), which will reduce roughness, increase the uniformity of the coating structure and improve their performance characteristics.

## Figures and Tables

**Table 1:** Chemical composition wt% of steel HS6-5-2 (1.3339): EN 4957-2000.

C	Si	Mn	P	S	Cr	Mo	W	V
0.8–0.88	max 0.45	max 0.4	max 0.03	max 0.03	3.8–4.5	4.7–5.2	5.9–6.7	1.7–2.1

**Table 2:** Operating modes of the “Elitron 52-A” unit.

Type of Generator	Capacitance, C, µF	Voltage, U, V	Frequency, Hz	Pulse Duration, s	Discharge Energy, Wp, J	Productivity, cm^2^/min
Transistor–thyristor (TT)	120	100	100	10^−7^–10^−8^	0.36	1.0–1.3
	300				0.90	

**Table 3:** Phase composition (wt%) of the alloying electrode.

WB_2_	ZrB_2_	Zr_3_O
72	23	5

**Table 4:** Qualitative parameters of coatings.

Discharge Energy, J	Roughness, µm		Strengthened Layer		Continuity, %
	Ra	Rz	HV	h, µm	
0.36	7.5146	42.8326	1125.9	10–20	75
0.90	7.7075	44.1509	1492.1	15–30	90

**Table 5:** Distribution of elements in the surface layer of the sample after ESA at Wp = 0.90 J.

** 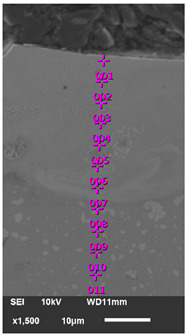 **	**Point**	**Elements Mass %**									
		**B**	**C**	**Cr**	**Zr**	**W**	**Mo**	**V**	**Ni**	**Fe**	**Total**
	1	5.78	1.42	1.06	11.69	57.46	1.07	0.09	0.16	21.27	100
	2	3.79	1.23	2.2	9.39	46.28	1.23	0.11	0.12	35.65	100
	3	4.38	1.35	2.51	6.58	31.85	2.05	0.06	0.21	51.01	100
	4	3.04	1.53	4.11	5.03	24.08	3.45	0.58	0.19	57.99	100
	5	4.37	1.24	3.15	4.67	43.26	2.32	0.65	0.15	40.19	100
	6	3.96	1.25	2.65	3.78	26.39	2.65	0.89	0.15	58.28	100
	7	2.18	1.29	2.77	1.76	23.27	3.74	0.76	0.27	63.96	100
	8	2.13	1.02	3.72	1.22	25.3	2.73	1.02	0.29	62.57	100
	9	1.78	0.9	3.58	0.24	13.24	3.56	1.14	0.6	74.96	100
	10	1.53	0.88	3.17	0.27	7.17	3.87	1.86	0.49	80.76	100
	11	0.55	0.97	3.13	0.21	5.12	4.72	1.72	0.68	82.9	100

**Table 6:** Roughness of samples for tribotechnical testing.

Samples	Roughness, µm	
	Ra	Rz
HS6-5-2 steel without ESA	1.19	8.00
ESA at Wp = 0.36 J	0.46	4.18
ESA at Wp = 0.90 J	0.49	3.61

**Table 7:** The average wear depth for each sample.

Samples	Average Wear Depth, μm
HS6-5-2 steel without ESA	1.52 ± 0.12
ESA at Wp = 0.36 J	0.88 ± 0.33
ESA at Wp = 0.90 J	0.69 ± 0.03

## Data Availability

The original contributions presented in this study are included in the article. Further inquiries can be directed to the corresponding author.

## References

[1] Eickelpasch N., Kalwa H., Steiner H., Priesmeyer U. (1997). The application of mechanical and thermal cutting tools for the dismantling of activated internals of the reactor pressure vessels in the Versuchsatomkraftwerk, Kahl and the Gundremmingen Unit A. Nucl. Eng. Des..

[2] Lee G.-R., Lim B.-J., Cho D.-W., Park C.-D. (2022). Selection methodology of the optimal cutting technology for dismantling of components in nuclear power plants. Ann. Nucl. Energy.

[3] Matteucci P., Cepolina F. (2015). A robotic cutting tool for contaminated structure maintenance and decommissioning. Autom. Constr..

[4] Rizzo A., Goel S., Grilli M.L., Iglesias R., Jaworska L., Lapkovskis V., Novak P., Postolnyi B.O., Valerini D. (2020). The Critical Raw Materials in Cutting Tools for Machining Applications: A Review. Materials.

[5] Pogrebnjak O.D., Dyadyura K.O., Gaponova O.P. (2015). Features of Thermodynamic Processes on Contact Surfaces of Multicomponent Nanocomposite Coatings with Hierarchical and Adaptive Behaviour. Metallofiz. Noveishie Tekhnol..

[6] Kang K., Su S., Yu B., Sun Z., Hu S., Wang Z., Zhao C., Wu L., Luo G., Wei R. (2025). The review and prospect of tool coating technology. Int. J. Adv. Manuf. Technol..

[7] Wang X.-X., Wang Y.-H., Ling Z.-C., Yuan Z.-P., Shi J.-J., Qin J., Sun H.-W., Pan K.-M., Geng Z.-M., Ma H.-L. (2025). Strategies for superhard tool coating materials: Focus on preparation methods and properties. J. Iron Steel Res. Int..

[8] Berladir K., Hovorun T., Botko F., Radchenko S., Oleshko O. (2025). Thin Modified Nitrided Layers of High-Speed Steels. Materials.

[9] Alves U.C., Ricci V.P., Mota I.G.C., Koga G.Y., Hassui A., Ventura C.E.H. (2025). Mechanical and tribological characterization of TiAlN/TiN and TiSiN/AlTiN coating systems for cutting tools. J. Manuf. Process..

[10] Drnovšek A., Rebelo de Figueiredo M., Vo H., Xia A., Vachhani S.J., Kolozsvári S., Hosemann P., Franz R. (2019). Correlating high temperature mechanical and tribological properties of CrAlN and CrAlSiN hard coatings. Surf. Coat. Technol..

[11] Twardowska A., Ślusarczyk Ł., Kowalski M. (2022). Impact of Deposition of the (TiB_x_/TiSi_y_C_z_) x3 Multilayer on M2 HSS on the Cutting Force Components and Temperature Generated in the Machined Area during the Milling of 316L Steel. Materials.

[12] Nunthavarawong P., Rangappa S.M., Siengchin S., Dohda K. (2022). Diamond-Like Carbon Coatings: Technologies and Applications.

[13] Liu X.-L., Lin Z., Zhao H.-J., Sun F. (2024). Study on Microstructure, Mechanical Properties, Tribological Properties and Service Performance of CrAlN and CrAlBN Coatings Deposited on Powder Metallurgy High-Speed Steel (PM-HSS) and Shaper Cutter by Arc Ion Plating Technique. Coatings.

[14] Tarelnyk V.B., Gaponova O.P., Konoplianchenko I.V., Tarelnyk N.V., Mikulina M.A., Gerasimenko V.A., Vasylenko O.O., Zubko V.M., Melnyk V.I. (2022). Properties of Surfaces Parts from X10CrNiTi18-10 Steel Operating in Conditions of Radiation Exposure Retailored by Electrospark Alloying. Pt. 3. X-ray Spectral Analysis of Retailored Coatings. Metallofiz. Noveishie Tekhnol..

[15] Chen L., Lan Y., Cheng Y., Zeng J., Ma Y., Yu S., Ding Z., Liu B., Zhang J., Peng H. (2024). Friction behavior and wear mechanism of laser cladded FeNiCr-WC composite coatings in comparison with different friction pairs. J. Mater. Res. Technol..

[16] Xian G., Xiong J., Fan H., Jiang F., Guo Z., Zhao H., Xian L., Jing Z., Liao J., Liu Y. (2022). Investigations on microstructure, mechanical and tribological properties of TiN coatings deposited on three different tool materials. Int. J. Refract. Met. Hard Mater..

[17] Wada T., Hanyu H. (2017). Tool wear of (Ti, Al) N-coated polycrystalline cubic boron nitride compact in cutting of hardened steel. IOP Conf. Ser. Mater. Sci. Eng..

[18] Sousa V.F.C., Silva F.J.G. (2020). Recent Advances in Turning Processes Using Coated Tools-A Comprehensive Review. Metals.

[19] Tan D.-W., Zhu L.-L., Wei W.-X., Yu J.-J., Zhou Y.-Z., Guo W.-M., Lin H.-T. (2020). Performance improvement of Si_3_N_4_ ceramic cutting tools by tailoring of phase composition and microstructure. Ceram. Int..

[20] Kumar B.P., Rao P.S., Ravi Kiran D.S.S., Venkatesh D.J., Rao C.V. (2025). Parametric optimization of Al_2_O_3_-ZrO_2_ (Y_2_O_3_) based self-lubricating composite cutting tool materials for turning operations using TOPSIS method. Int. J. Interact. Des. Manuf..

[21] Manokhin A., Klymenko S., Beresnev V., Zakiev V., Klymenko S., Tonkonogyi V., Ivanov V., Trojanowska J., Oborskyi G., Edl M., Kuric I., Pavlenko I., Dasic P. (2020). To the Question of the Mechanism of the Effect of Coating on the Durability of Tools from PCBN. Advanced Manufacturing Processes. InterPartner 2019.

[22] AbuAlRoos N.J., Azman M.N., Baharul Amin N.A., Zainon R. (2020). Tungsten-based material as promising new lead-free gamma radiation shielding material in nuclear medicine. Phys. Medica.

[23] Windsor C.G., Astbury J.O., Davidson J.J., McFadzean C.J.R., Morgan J.G., Wilson C.L., Humphry-Baker S.A. (2021). Tungsten boride shields in a spherical tokamak fusion power plant. Nucl. Fusion.

[24] Liu Y., Liu X., Lai C., Ma J., Meng X., Zhang L., Xu G., Lu Y., Li H., Wang J. (2023). Boriding of tungsten by the powder-pack process: Phase formation, growth kinetics and enhanced neutron shielding. Int. J. Refract. Met. Hard Mater..

[25] McAlister D. (2016). Neutron Shielding Materials.

[26] Windsor C.G., Astbury J.O., Morgan J.G., Wilson C.L., Humphry-Baker S.A. (2022). Activation and transmutation of tungsten boride shields in a spherical tokamak. Nucl. Fusion.

[27] Lin Y., McFadzean C., Humphry-Baker S.A. (2022). Oxidation resistance of WB and W_2_B-W neutron shields. J. Nucl. Mater..

[28] Pangilinan L.E., Turner C.L., Akopov G., Anderson M., Mohammadi R., Kaner R.B. (2018). Superhard Tungsten Diboride-Based Solid Solutions. Inorg. Chem..

[29] Akopov G., Yeung M.T., Turner C.L., Mohammadi R., Kaner R.B. (2016). Extrinsic hardening of superhard tungsten tetraboride alloys with group 4 transition metals. J. Am. Chem. Soc..

[30] Mościcki T., Chrzanowska-Giżyńska J., Psiuk R., Denis P., Mulewska K., Kurpaska Ł., Chmielewski M., Wiśniewska M., Garbiec D. (2022). Thermal and mechanical properties of (W,Zr)B_2−z_ coatings deposited by RF magnetron sputtering method. Int. J. Refract. Met. Hard Mater..

[31] Mościcki T., Psiuk R., Radziejewska J., Wiśniewska M., Garbiec D. (2021). Properties of spark plasma sintered compacts and magnetron sputtered coatings made from Cr, Mo, Re and Zr alloyed tungsten diboride. Coatings.

[32] Khor K., Yu L., Sundararajan G. (2005). Formation of hard tungsten boride layer by spark plasma sintering boriding. Thin Solid Films.

[33] Maździarz M., Psiuk R., Krawczyńska A., Lewandowska M., Mościcki T. (2022). Effect of zirconium doping on the mechanical properties of W_1−x_ZrxB_2_ on the basis of first-principles calculations and magnetron sputtered films. Arch. Civ. Mech. Eng..

[34] Psiuk R., Milczarek M., Jenczyk P., Denis P., Jarząbek D.M., Bazarnik P., Pisarek M., Mościcki T. (2021). Improved mechanical properties of W-Zr-B coatings deposited by hybrid RF magnetron–PLD method. Appl. Surf. Sci..

[35] Rzempołuch J., Stasiak T., Maździarz M., Jasiński J., Woy U., Psiuk R., Kowal M., Kosińska A., Wilczopolska M., Mulewska K. (2025). Characterization of He⁺ implanted W-Zr-B thin films deposited by HiPIMS on additively manufactured Inconel 617 as a candidate system for nuclear components. J. Nucl. Mater..

[36] Storozhenko M.S., Umanskyi O.P., Tarelnyk V.B., Koval O.Y., Gubin Y.V., Mikulina M.O., Martsenyuk I.S., Kostenko O.D., Kurinna T.V. (2020). Structure and Wear Resistance of FeNiCrBSiC–MeB_2_ Electrospark Coatings. Powder Metall. Met. Ceram..

[37] Benkovsky I., Tsyntsaru N., Silkin S., Petrenko V., Pakstas V., Cesiulis H., Dikusar A. (2023). Synthesis, Wear and Corrosion of Novel Electrospark and Electrospark–Electrochemical Hybrid Coatings Based on Carbon Steels. Lubricants.

[38] Gaponova O.P., Tarelnyk V.B., Tarelnyk N.V., Myslyvchenko O.M. (2023). Nanostructuring of Metallic Surfaces by Electrospark Alloying Method. JOM.

[39] Tarelnyk V.B., Gaponova O.P., Konoplianchenko Y.V., Martsynkovskyy V.S., Tarelnyk N.V., Vasylenko O.O. (2019). Improvement of Quality of the Surface Electroerosive Alloyed Layers by the Combined Coatings and the Surface Plastic Deformation. III. The Influence of the Main Technological Parameters on Microgeometry, Structure and Properties of Electrolytic Erosion Coatings. Metallofiz. Noveishie Tekhnol..

[40] Garbiec D., Wiśniewska M., Psiuk R., Denis P., Levintant-Zayonts N., Leshchynsky V., Rubach R., Mościcki T. (2021). Zirconium alloyed tungsten borides synthesized by spark plasma sintering. Arch. Civ. Mech. Eng..

[41] Vander Voort G.F. (2004). Metallography and Microstructures.

[42] Gaponova O.P., Tarelnyk V.B., Martsynkovskyy V.S., Konoplianchenko I.V., Melnyk V.I., Vlasovets V.M., Sarzhanov O.A., Tarelnyk N.V., Mikulina M.O., Polyvanyi A.D. (2021). Combined Electrospark Running-in Coatings of Bronze Parts. Part 1. Structure and Mechanical Properties. Metallofiz. Noveishie Tekhnol..

[43] Haponova O., Tarelnyk V., Mościcki T., Tarelnyk N. (2025). Hybrid Surface Treatment Technologies Based on the Electrospark Alloying Method: A Review. Coatings.

[44] Kumar A., Batham H., Das A.K. (2021). Microhardness of Fe-TiB2 composite coating on AISI 304 stainless steel by TIG coating technique. Mater. Today Proc..

[45] Zhao S., Xu S., Yang L., Huang Y. (2022). WC-Fe metal-matrix composite coatings fabricated by laser wire cladding. J. Mater. Process. Tech..

[46] Penyashki T., Kostadinov G., Kandeva M. (2025). Improvement of Surface Properties of Carbon Steel Through Electrospark Coatings from Multicomponent Hard Alloys. Materials.

[47] Tarelnyk V., Konoplianchenko I., Gaponova O., Tarelnyk N., Martsynkovskyy V., Sarzhanov B., Sarzhanov O., Antoszewski B. (2020). Effect of Laser Processing on the Qualitative Parameters of Protective Abrasion-Resistant Coatings. Powder Metall. Met. Ceram..

[48] Ciski A. (2018). Deep Cryogenic Treatment and Tempering at Different Temperatures of HS6-5-2 High Speed Steel. Arch. Metall. Mater..

